# Salvage of stoma stenosis using a fenestrated ultrathin ALT perforator flap following Kock pouch ileostomy

**DOI:** 10.1080/23320885.2025.2479007

**Published:** 2025-03-14

**Authors:** Evelyne Smith, Pietro Di Summa, Dieter Hahnloser

**Affiliations:** ^a^ Department of Visceral Surgery, Lausanne University Hospital CHUV and University of Lausanne UNIL, Lausanne, Switzerland; ^b^ Department of Plastic and Hand Surgery, Lausanne University Hospital (CHUV), Lausanne, Switzerland

**Keywords:** Abdominal wall reconstruction, stoma transposition, short bowel syndrome, kock pouch, anterolateral thigh flap

## Abstract

Abdominal wall defects can present a challenge for reconstructive surgeons, especially in the presence of major visceral issues. Anterolateral thigh flaps (ALT) are widely used in complex abdominal wall repair, whether pedicled or free, innervated, or denervated. Previous reports primarily focus on reconstruction of the outer layers of the abdominal wall (muscle, fascia, and skin); however, this case highlights the potential of ALT flaps to address complications at the mucosa-skin interface, particularly stomal issues. This case report aims to demonstrate the versatility of the ALT flap at the mucosa-skin contact area, specifically to salvage a stenotic stoma following a Kock pouch (K-pouch) ileostomy. The patient developed multiple complications, including stomal retraction, stenosis, and leakage, which required innovative reconstructive techniques. Here, transposition of a fenestrated ultrathin pedicled ALT flap was used to reconstruct the abdominal wall while accommodating a retracted stoma in the middle of the skin paddle, restoring functional defecation. Initial postoperative complications, such as stomal leak and partial flap dehiscence, were managed with secondary flap adjustments, resulting in long-term successful outcomes. At the two year of follow-up, the stoma remained functional, with no recurrence of stenosis or skin irritation, and the donor site healed without morbidity [1].

## Case report

This case involves a 63-year-old man with Crohn’s disease complicated by abdominal and perineal fistulas, as well as rectal adenocarcinoma. The patient initially underwent a low anterior resection with a low anastomosis. This procedure, typically performed for rectal cancer, involves removing the rectum and part of the sigmoid colon, followed by creating an anastomosis between the remaining colon and the rectum or the anus to preserve bowel function. The term ‘low’ refers to the proximity of the anastomosis to the anus.

Post-operatively, the patient experienced complications that led to additional surgical interventions. When such reconstructions fail or complications arise, more extensive procedures, such as enterectomies with anastomosis and proctocolectomy, may be required, particularly in cases involving inflammatory bowel disease or colorectal cancer. A ‘proctocolectomy’ is the surgical removal of the rectum and the entire colon, often performed for conditions like inflammatory bowel disease or colorectal cancer. In this case, the patient underwent multiple enterectomies and colonic resections, which resulted in short bowel syndrome (SBS) and the creation of an ileostomy.

As a result of SBS, the patient experienced severe diarrhea, weight loss (BMI: 19 kg/m^2^), and malnutrition, demonstrated by low albumin (26 g/L) and pre-albumin (0.12 g/L) levels. He further required multiple stoma reconstructions. Eventually, the patient underwent a total proctocolectomy with the creation of a permanent terminal ileostomy using a Kock pouch (K-pouch). When sufficient small bowel remains following a proctocolectomy, an ileal pouch with an anal anastomosis (called a ‘J-pouch’) is typically the preferred option. A J-pouch is constructed by folding the small intestine into a ‘J’ shape to create an internal reservoir, connected directly to the anus, allowing for stool to pass naturally. If there is an insufficient amount of small bowel, or the anal sphincters are too weak, a continent ileostomy like a K-pouch is the last resort. The K-pouch is an internal reservoir constructed from the small intestine, allowing waste to be drained periodically *via* self-catheterization through a stoma. This approach eliminates the need for an external ostomy bag but still requires a permanent stoma.

**Figure 1.**
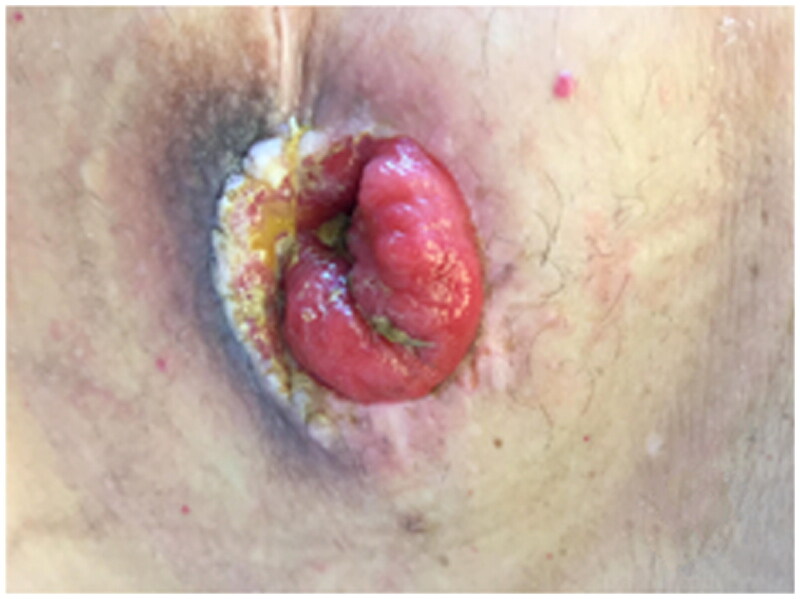
Represents the pre-operative status of the stoma following the creation of a Kock pouch in the left inferior abdominal quadrant. The figure shows a hyper-granulated stoma surrounded by fibrotic and constrictive tissues. This state occurred due to complications of the stomal retraction, stenosis, and skin irritation from chronic stool exposure. The tissue surrounding the stoma appears inflamed and shows hyper-granulation, highlighting the challenges in maintaining the integrity of the mucosa-skin interface before reconstructive surgery using the fenestrated ultrathin ALT perforator flap.

After the construction of a K-pouch, the patient developed multiple episodes of stomal retraction and stenosis because of fascial narrowing and skin irritation due to chronic exposure to stool caused by the short bowel syndrome. Initial management, involving peri-stomal cutaneous and fascial excisions to widen the orifice, which were insufficient. The stoma became painful, accompanied by skin retraction and a hyper-granulating mucosa (Figure 1).

The multidisciplinary surgical plan included reconstructing the skin around the stoma and reducing the tension at the stoma level, with healthy vascularized skin meeting the mucous membrane of the stoma to prevent stenosis. A pedicled ultrathin anterolateral thigh (ALT) flap was selected as the optimal choice for the stoma site reconstruction. This technique allowed the flap to be transposed while maintaining its pedicled blood supply, ensuring adequate vascularization. The flexibility to raise the flap on different perforators facilitated the creation of a precise stoma opening. This approach minimized donor site morbidity and reduced the risk of complications at the harvest site.

In this case, alternative reconstructive options, such as a free flap, were not considered because of specific factors related to the defect size, patient condition, and unique requirements of this stoma reconstruction. Free flaps are often effective for treating smaller defects. However, in this patient, the defect was extensive and required robust, well-vascularized tissue to reconstruct both the stoma and the surrounding skin complications. The pedicled ALT flap was chosen because it provided a reliable vascular supply, which is crucial for enhancing blood flow to the stoma in order to minimize the risk of stoma necrosis and stenosis, which are common complications in patients with a chronic stoma.

The pedicled ALT flap eliminated the need for microsurgical anastomosis, reduced the operative time and the surgical complexity in a patient already compromised by malnutrition, Crohn’s disease, and its sequelae. Its versatility allowed for tension-free closure and incorporation of a stoma while providing a durable and vascularized coverage at the reconstruction site - reinforcing its advantages over a free flap. This approach effectively balanced the goals of minimizing donor site morbidity while addressing the patient’s functional and reconstructive needs.

### Surgical technique

**Figure 2.**
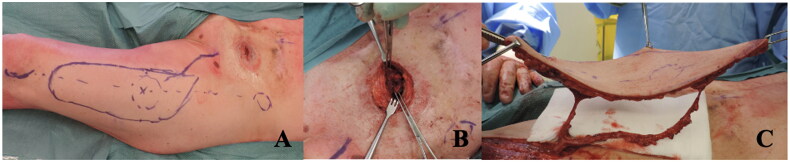
Illustrates the surgical planning and operative process of the reconstruction using the fenestrated ultrathin ALT perforator flap. Panel A: the preoperative marking on the left thigh outlines the design of the ALT flap. This includes markings for the location of the perforator vessels (proximal-P2 and distal-P1) that will supply vascularization to the flap. The elliptical fenestration in the flap design is intended to accommodate the stoma site after transposition. These markings reflect careful planning to ensure adequate vascularization and the ability to integrate the flap into the defect without excessive tension. Panel B: the intraoperative view of the stoma site shows the preparation and dissection of the intestinal mucosa. The scarred and retracted tissue around the stoma was excised with a 3 cm margin. This step is crucial for separating the intestinal mucosa from surrounding scar tissue and creating a clean field for integration with the flap. Panel C: the harvested ALT flap, vascularized by two perforator vessels (P1 and P2), is shown. The flap includes an elliptical fenestration that will accommodate the stoma, which is transposed into the abdominal wall defect.

**Figure 3.**
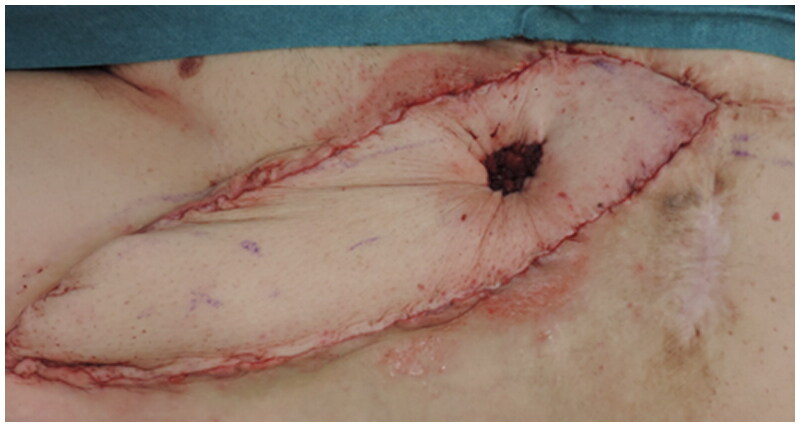
Postoperative status. The ALT flap is harvested and fenestrated to accommodate the stoma with a tension-free coverage with sutures, securing the mucosa to the flap to ensure a functional and leak-proof stoma – a successful initial outcome with no apparent signs of ischemia or necrosis of the flap.

The procedure began with excision of all the skin around the stoma, maintaining a 3 cm margin. While the colorectal surgeons dissected the intestinal mucosa from the scarred skin tissue, the plastic surgery team simultaneously harvested the ALT flap from the left thigh. The flap was isolated with two perforating vessels, one proximal (P2) and one distal (P1) to the designed fenestration (Figure 2). The flap was passed under the rectus femoris and sartorius muscles and rotated 180° into the defect, as previously described [2]. An elliptical fenestration was created through the flap between the two perforator vessels (P1 and P2), marking the new stoma site. The intestinal mucosa was brought through the flap and sutured to the de-epithelialized skin using polydioxanone (Ethicon PDS^®^ II) 4-0 monofilament absorbable sutures. (Figure 3) The rest of the flap and the donor site were closed on two layers with polyglactin 910 (Ethicon Vycril^®^) 2.0 braided absorbable suture followed by a poliglecaprone 25 (Ethicon monocryl^®^) 3.0 monofilament synthetic absorbable suture at the skin level.

### Outcomes and complications

**Diagram 1.**
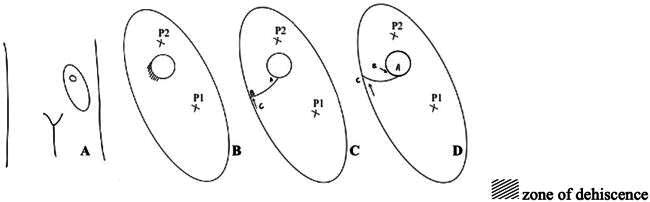
Panel A: Depicts the post-operative location of the stoma with the adjustments of the fenestrated ultrathin ALT flap in the left lower quadrant of the abdomen. The elliptical area represents the transferred ultrathin ALT flap, and the circular area is the fenestrated portion of the flap housing the stoma. Panel B: Enlarged version of the reconstructed area. The harvested ALT flap is illustrated with two perforator vessels (P1 and P2). The fenestration, housing the stoma, is shown centrally between these perforator vessels. The hatched area represents the zone of dehiscence, highlighting a complication requiring secondary adjustments to ensure the functionality of the stoma and the integration of the flap. Panel C: Demonstrates the surgical adjustments made to address the dehiscence and ensure proper alignment of the flap with the stoma. The line from the stoma marks the separation of the distal portion of the flap, supported by the perforator P2. Panel D: the flap is adjusted, with the distal portion being repositioned deeper into the abdominal cavity.

The initial flap perfusion was satisfactory; however, on the third postoperative day, the patient developed a stomal leak leading to stercoral contamination of the flap. The leak was aggravated by diluted fecal material due to SBS, leading to dehiscence of over a quarter of the lateral and inferior borders of the flap, along with retraction of the stoma orifice. Following multiple surgical washouts and local debridements of the stoma site, an additional flap division was performed based on the distal perforator (P2). This allowed the mobilization of a portion of the flap deeper into the stoma and abdominal cavity, which facilitated the final closure of the stoma-skin interface and prevented further fecal leakage (Diagram 1).

In the visceral surgery department, management of high-output ileostomies requires a comprehensive approach to maintain hydration, electrolyte balance, and proper intestinal function. Ensuring adequate hydration is a cornerstone of care, as individuals with high-output ileostomies are at an increased risk of dehydration and electrolyte depletion. This usually involves the consumption of oral rehydration solutions with balanced sodium and potassium. However, in cases of severe dehydration or limited oral intake, intravenous fluids and electrolytes may be necessary to restore balance effectively.

The administration of proton pump inhibitors can reduce gastric acid secretion, thereby minimizing fluid loss through an ileostomy. Additional therapies, such as cholestyramine (Quantalan) to bind bile acids, pancreatic enzyme supplements such as Creon to optimize nutrient absorption, and loperamide to slow intestinal transit, can significantly decrease stoma output. Probiotics such as Lactiplant may also support the gut microbiota balance. In this situation, reducing the high stoma output not only prevented dehydration but also played a critical role in minimizing the risk of contamination at the flap-mucosa stage, a key concern in surgical recovery.

## Follow-up period

At the 8-week follow-up with the general surgery team, the patient was able to empty the K-pouch without para-stomal leakage or signs of recurrence of stenosis. No complications were observed at the harvest site.

**Figure 4.**
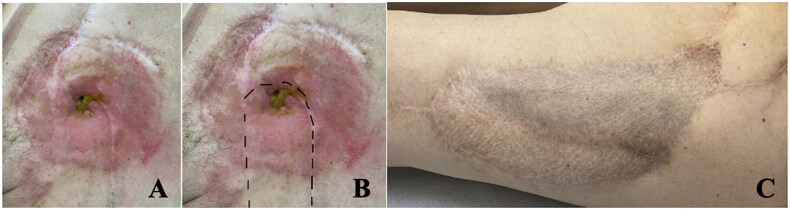
Demonstrates long-term outcomes following reconstruction. Panel A: 2-year post-operative appearance of the stoma with a successful integration of the flap with the stoma. The surrounding skin appears healthy, with no signs of stenosis, necrosis, or significant irritation. The stoma is functional; the mucosa-skin-interface is well aligned. Panel B: Overlays a dashed outline to highlight the surgical reconstruction area with the positioning of the flap. Panel C: Illustrates the donor site on the left thigh, demonstrating favorable healing with no significant morbidity.

At 2 years post-surgical intervention, no donor site morbidity was observed, and favorable evolution with the stoma maintaining functionality with no signs of skin excoriation or stenosis. (Figure 4)

## Discussion

Stoma formation is one of the most commonly performed surgical procedures in colorectal surgery for the surgical treatment of malignant and inflammatory bowel diseases. There are various types of stomas. There is a lifetime risk of complications associated with stoma formation; the incidence of complications is greatest in the first five years following surgery. The most common early complications include infection, improper location, skin excoriation, leakage, dehydration, retraction, and necrosis of the stoma. Late complications include para-stomal hernia, stomal prolapse, stenosis, and peristomal dermatitis [3].

Skin excoriation and painful stomal stenosis are traditionally treated by resection of the dysfunctional K-pouch and construction of a new one with the remaining small intestine, along with widening of the fascial opening of the stoma. Unfortunately, such a procedure was contraindicated in our patient due to multiple laparotomies, intestinal resections resulting in SBS, and significant intra-abdominal adhesions. Other loco-regional reconstructive options, such as alternative pedicle flaps or direct closure with abdominal wall component separation, were considered. However, direct closure with component separation was not advised in this case because of the existing stoma and extensive prior abdominal surgeries, which compromised the integrity and mobility of the abdominal wall. Additionally, the degree of intraperitoneal adhesions and the risk of ischemic complications further limited the feasibility of fascial mobilization.

The decision-making process in flap selection requires careful consideration of both the anatomical constraints and functional requirements of the reconstruction. Given the location of the abdominal wall defect inferior to the umbilicus, the ALT flap was deemed the most reliable and versatile option for reconstruction, offering robust vascularity and adaptability to the specific requirements of the defect. To confirm the feasibility of the ALT flap and to optimize planning, an angio-CT was performed, which visualized the ALT pedicle root and identified the course and caliber of the perforators. This imaging was fundamental in determining the precise location for flap harvest, ensuring adequate vascularization, and minimizing the risk of ischemic complications during or after surgery.

In recurrent infections, radiated or multi-operated sites, vascularized flaps facilitate effective antibiotic delivery and wound healing with vascularized tissue. The ALT flap can be transferred as a free flap for reconstruction of the upper abdominal quadrants, while it can be kept pedicled for the para-umbilical and infra-umbilical regions. The thigh-to-trunk length ratio limits the reaching level when flaps are pedicled. In this case, the ALT flap was selected not only for its anatomical suitability but also for its ability to address the specific challenges. These included poor skin compliance, limited bowel mobility, and a high risk of wound dehiscence due to previous surgeries. Alternative vascularized options, such as rectus abdominis or omental flaps, were considered; however, these were deemed less favorable because of the extensive prior use of the abdominal wall musculature and the potential for further weakening of abdominal support.

During planning and execution, one of the main challenges was to ensure tension-free integration of the flap with the surrounding tissues, given the complex geometry of the defect and the need to accommodate the stoma within the flap. Postoperatively, the patient experienced a minor stoma leakage complication that required secondary modifications. This was managed effectively with the dual-perforator construct of the flap, which allowed for separation and repositioning to reduce tension and ensure definitive healing. Despite these challenges, the preserved vascularization and thin profile of the flap facilitated healing and reduced the risk of infection, especially in contamination-prone areas such as the stoma.

For abdominal wall reconstruction, the ALT flap provides remarkable versatility and adaptability. They can be harvested as composite flaps, along with the vastus lateralis muscle, allowing dynamic tissue function when reinnervated [4]. For superficial resurfacing, the ALT flap can be raised just under the level of Scarpa’s fascia as an ultrathin flap [5], as in the present report.

The use of ultrathin flap provides distinct advantages over conventional thickness flaps. Raising the flap just beneath the Scarpa fascia allows for improved contouring to match the surrounding abdominal and stomal tissues, reducing bulkiness and minimizing discomfort or functional restrictions. The reduced thickness also facilitates tension-free integration with surrounding tissues, reducing mechanical strain at the stoma-skin interface, which is a critical factor in preventing complications such as necrosis, stenosis, or impaired healing. The preserved vascularization closer to the surface enhanced healing and minimized complications from chronic fecal contamination. The thinner flap contributed to better outcomes by enhancing aesthetic results and reducing donor-site morbidity. Less invasive harvesting techniques specific to the ALT flap not only minimize postoperative pain but also accelerate recovery, ultimately improving the patient’s quality of life. Trans-flap stoma integration has previously been explored as a viable approach for directing stoma effluent in patients with limited skin compliance and restricted bowel mobilization following complex abdominal wall procedures, offering a low-risk solution with minimal complications [6,7].

In this case, the flap was harvested with two different perforator vessels, allowing adequate vascularization throughout the flap, even with the stoma outlet. Moreover, this dual-perforator construct facilitated secondary modifications when stoma leakage occurred. The distal portion of the flap, based on the second perforator, was further separated to follow the stoma deeper into the abdominal cavity, thereby reducing tension on the stoma-skin contact surface and ensuring definitive healing. The thin profile of the flap enabled effective trans-flap stoma housing, ensuring optimal perfusion, and addressing challenges such as poor skin compliance or limited bowel mobility due to prior operations.

This case report further highlights the versatile possibilities of tailoring ultrathin ALT flaps in reconstructive surgery. They not only allow for the exit of the stoma within the flap itself, but also guarantee optimal perfusion, resist gross fecal contamination, and permit further division or transposition if required. The combination of these features underscores the superior outcomes associated with ultrathin flaps, particularly in complex cases of abdominal wall and stomal reconstructions.
